# Correlation between choroidal vascularity and retrobulbar ocular blood flow changes and thyroid-associated ophthalmopathy activity: a cross-sectional study

**DOI:** 10.1186/s12886-024-03308-w

**Published:** 2024-02-13

**Authors:** Xinghong Sun, Mengru Su, Xiaowen Zhang, Haiyun Shen, Zhenggao Xie, Wentao Kong, Dandan Zhu

**Affiliations:** 1 Department of Ophthalmology, Nanjing Drum Tower Hospital, The Affiliated Hospital of Nanjing University Medical School, No.321, Zhongshan Road, Gulou District, Nanjing, 210000 China; 2 Department of Endocrine, Nanjing Drum Tower Hospital, The Affiliated Hospital of Nanjing University Medical School, Nanjing, China; 3 Department of Ultrasonography, Nanjing Drum Tower Hospital, The Affiliated Hospital of Nanjing University Medical School, No.321, Zhongshan Road, Gulou District, Nanjing, 210000 China

**Keywords:** Choroidal vascularity, Thyroid-associated orbitopathy, Color doppler imaging, Optical coherence tomography angiography

## Abstract

**Objective:**

To evaluate the alterations in retrobulbar color Doppler imaging (CDI) parameters and retinal/choroidal optical coherence tomography angiography (OCTA) parameters and their association with the clinical activity and severity in thyroid-associated orbitopathy (TAO) patients.

**Methods:**

In this study, the retrobulbar flow parameters including resistance index (RI), Pulsatile Index(PI), peak systolic velocity (PSV) and end diastolic velocity (EDV) in posterior ciliary artery (PCA), central retinal artery (CRA) and ophthalmic artery (OA) were determined by CDI. Moreover, the retina and choroidal vascularity including the superficial vessel density (SVD), deep vessel density (DVD), choroidal thickness (ChT) and choroidal vascularity, including total choroidal area (TCA), luminal area (LA), stromal area (SA) and Choroidal Vascularity Index (CVI), were determined by OCTA. All patients grouped as active TAO and inactive TAO based on Clinical activity score (CAS). We picked the severe eye among the subjects and compared all parameters between two groups. We analyzed the correlations among those parameters.

**Results:**

There was a significant difference in CAS score, proptosis value, ChT, LA, CVI between patients with active TAO and inactive TAO. In the active group, PSV and EDV of PCA were significantly higher than the inactive group. On logistic regression analysis, CAS was closely associated with PSV-PCA. On multiple linear regression, proptosis value was closely associated with ChT, LA, SA and CVI.

**Conclusion:**

Choroidal vascularization and retrobulbar blood flow were concurrently higher in active TAO patients and several variables in choroid circulation was closely related to TAO clinical features.

## Introduction

Thyroid-associated orbitopathy (TAO) is an autoimmune inflammatory disorder which is usually characterized by ocular manifestations ranging from mild dryness, conjunctival hyperemia, chemosis, eyelid edema/retraction, and proptosis to severe forms of optic neuropathy and corneal ulceration [1]. A multicenter study found that 75% of TAO patients had active soft tissue involvement with upper eyelid swelling, while 50% had extraocular muscle involvement, and 25% had severe eye signs such as lagophthalmos and keratopathy [2].

Currently, the histological characteristics of TAO can be explained by increased infiltration of inflammatory cells and proliferation of perimysial fibroblasts [3, 4]. As the degree of extracellular matrix deposition increases, the rectus muscle and soft orbital tissue expand. Previous studies have reported that hemodynamic changes in ocular blood flow may play a vital role in the pathophysiology of uncontrolled orbital tissues proliferation [5].

The choroid vasculature is generally regarding as the major blood supply for the outer retina. In addition to its blood supply function, this vascular network contains a variety of secretory cells including melanocytes, fibroblasts, and resident immunocompetent cells [6]. Other possible functions of the choroid include action as a heat source for the outer retina, regulation of osmotically active molecule synthesis, and modulation of intraocular pressure (IOP) [7, 8]. Thus, the possibility that the choroid is an ocular pathological sensory tissue should be assessed in depth.

Several non-invasive methods have been used to determine ocular blood flow in patients with TAO [9, 10]. Combining multiple methods to study TAO may provide a new way to figure out its pathophysiological features. Color Doppler imaging (CDI) has been increasingly applied for analysis of retrobulbar arterial vascular structures because of its high repeatability and quantifiable diagnostic function [11, 12]. Recently, Optical Coherence Tomography Angiography(OCTA) has been shown to be a fast and non-invasive diagnostic tool in the evaluation of retinal multilayer vasculature [1]. The choroid supplies the outer retina with oxygen and nourishment, which indicates that alterations in retinal vascularity may indirectly reflect choroidal flow changes. However, these comprehensive ocular vascularity alterations have been poorly investigated, and data regarding the relation between these factors in TAO patients are conflicting.

This study aimed to evaluate the changes in retroocular arterial structure and macular vascular flow in TAO using CDI and OCTA. We further analyzed the correlation of these hemodynamic parameters in patients with TAO.

## Materials and methods

The study was conducted at Affiliated Drum Tower Hospital of Nanjing University Medical School. Each patient provided informed consent in accordance with the Declaration of Helsinki, and the study was approved by the Nanjing Drum Tower Hospital Ethics Committee. Subjects first diagnosed with TAO were enrolled from August 2021 and September 2022.

All patients underwent a comprehensive ophthalmological examination, including visual acuity, slit lamp biomicroscopy, intraocular pressure examination. Ocular proptosis was measured using a Hertel exophthalmometer.

TAO activity was decided by the Clinical activity score (CAS), patients were classified as inactive disease(CAS < 3) or active disease (CAS ≥ 3).Subjects who had a history of prior injuries, optic neuropathy, other inflammatory disorders of uncertain cause, past orbital irradiation or surgery, and prior immunosuppressing therapies with steroids were excluded from this study. We studied the more severe eye.

### Optical coherence tomography angiography

**Fig.1:**
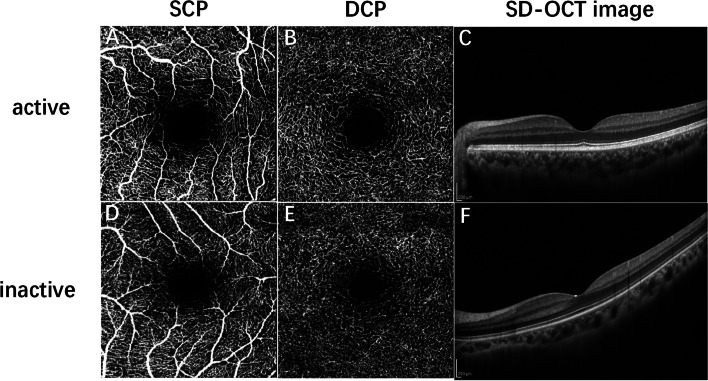
OCTA and SD- OCT images of TAO. **A** OCTA image of SCP in active eye. **B** OCTA image of DCP in active eye. **C** SD- OCT image of macular center in active eye. **D** OCTA image of SCP in inactive eye. **E** OCTA image of DCP in inactive eye. **F** SD- OCT image of macular center in inactive eye. SCP:superficial capillary plexus; DCP: deep capillary plexus; OCTA: optical coherence tomography angiography; SD- OCT: spectral- domain optical coherence tomograph

The retinal microvasculature was assessed using a single OCTA system (Spectralis OCT, Heidelberg Engineering, Heidelberg, Germany). Each patient underwent a 3.0*3.0 mm scan centered on the fovea. The scan was composed of 256 B-scans at a distance of 11 μm each, each B scan included 512 A scans and a single B-scan withan average of 5 frames.The superficial capillary plexus(SCP) and deep capillary plexus (DCP) were automatically segmented, SCP was defined from the inner limiting membrane(ILM) to the inner plexiform layer (IPL), and the DCP was defined from the IPL to the outer plexiform layer(OPL) (Fig. 1). The en-face images of SCP and DCP were exported and then imported into the ImageJ (National Institutes of Health, Bethesda, MD, USA). As previously described,the superficial and deep vessel density were calculated in the 3*3 mm area by the binarized images [13].

The choroidal vascular parameters were obtained using line scanning mode. The single line pattern scan centered on fovea using the EDI mode of a spectral-domain OCT(SD-OCT) device (Spectralis OCT; Heidelberg Engineering, Germany). The line was 30° in length and was captured in the high-resolution scanning mode. We acquired all OCT scans in the afternoon from 3 to 5 o'clock to control for the diurnal variation of choroidal.

**Fig.2:**
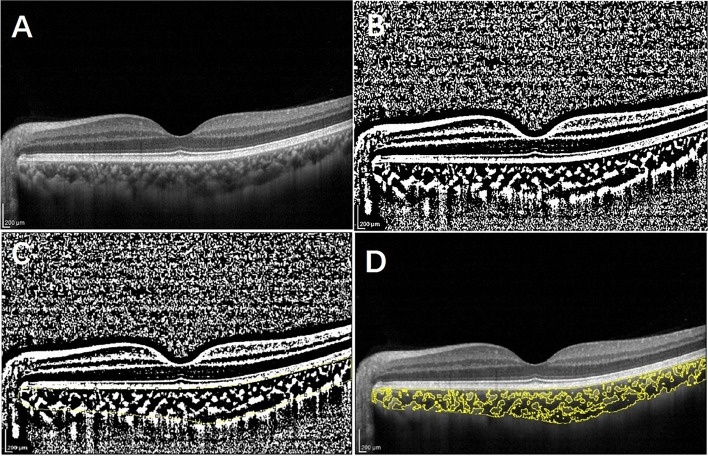
Measurement technique of choroidal vascularity index (CVI). **A **The enhanced depth optical coherence tomography(EDI-OCT) images of the subjects. **B **The binarisation image by Niblack auto local threshold method. **C** The total choroidal area(TCA) was determined using the region of interest (ROI) manager. **D **the color threshold tool was used to select the dark pixels, representing the luminal areas(LA).CVI was calculated by dividing LA to TCA

We manually removed the images with unclear choroidal scleral interface so as to maintain the better quality. All images were analyzed using the ImageJ (National Institutes of Health, Bethesda, MD, USA). Firstly, the image was binarized according to Niblack auto-local threshold, then we used the polygon tool to select the total choroidal region from the retinal pigment epithelium to the choroid-scleral junction and added it to the ROI manager. Then we convert the image to RGB format, and the luminal area (LA) was determined using color threshold tool. Finally, the total choroidal area (TCA) and LA were automatically calculated, Choroidal Vascularity Index (CVI) value was determined as the ratio of LA to TCA (Fig. 2).

The choroidal thickness (ChT) was considered as the vertical distance from the Bruch membrane to the choroid-scleral junction in the sub-fovea. It was manually measured by two independent examiners.

### Color doppler imaging measurements

The blood flow of retrobulbar vessels including ophthalmic artery (OA), central retinal artery (CRA) and posterior ciliary artery (PCA) was evaluated using the LOGIQ E9 and E20 ultrasound scanner (GE Healthcare, Chicago, III). The 9.0- MHz linear probe was applied for the ophthalmic artery, and the 12.0- MHz linear probe for the central retinal artery and posterior ciliary artery.

All patients were examined by an experienced sonographer with supine position. The measurement was performed according to the CDI measurement protocol [14]. All parameters were measured three times, and the mean results were used to analyze.

**Fig.3:**
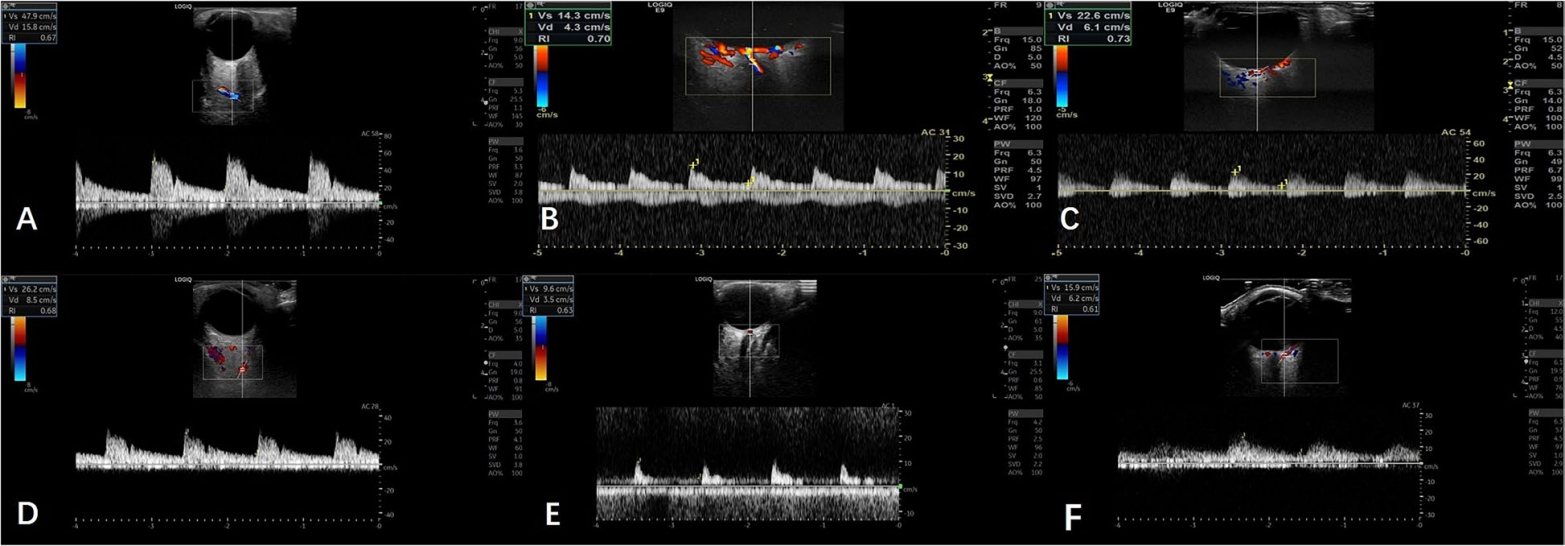
Color doppler ultrasound images of **A** ophthalmic artery, **B** central retinal artery, **C **posterior ciliary artery in active eyes. Color doppler ultrasound images of **D** ophthalmic artery, **E** central retinal artery, **F **posterior ciliary artery in non-active eyes

We acquired three parameters of each vessel:The peak systolic velocity (PSV), end diastolic velocity (EDV) and Pulsatile Index (PI) in each vessel were determined. Resistance Index (RI) = (PSV − EDV)/PSV, it is the most reproducible parameter of orbital blood flow to evaluate the peripheral vascular resistance [15] (Fig. 3).

## Statistical analysis

Statistical assessments in this research were performed using SPSS22.0. Descriptions of numerical data were expressed as mean ± standard deviations (SDs), categorical variables as numbers with percentages. Differences between inactive and active groups were compared using the two- sample t- test or Wilcoxon signed- rank test and χ2 test. The correlation degree and statistical significance between variables were analyzed by the Pearson correlation test. Logistic regression analysis was used to evaluate the relationships between CAS score and the OCTA/CDI parameters. Multiple linear regression analysis was used to evaluate the association between proptosis measurement of the study eye and the OCTA/CDI parameters. *P* value < 0.05 was considered statistically significant.

## Results

This study enrolled 51 TAO patients with a mean age of 49.24 ± 9.75 (range: 26–72 years). For patients with CAS < 3, the mean proptosis value was 16.72 ± 1.37 mm, while that of patients with CAS ≥ 3 was 20.65 ± 2.04. Age, sex distribution, BCVA, IOP, and TAO history were statistically similar between the two groups. However, there were significant differences between the two groups with asymmetry in terms of CAS score and proptosis value (Table 1).

**Table 1:** Baseline characteristics in inactive TAO patients and active TAO patients

Variables	**inactive TAO**	**active TAO**	***P* value**
	***n*=25**	***n*=26**	
**Gender(M/F)**	11/14	12/14	0.13
**Age (years)**	49.36±11.25	49.12±8.30	0.85
**BCVA (logMAR)**	0.0(0.0,0.2)	0.0(0.0,0.2)	0.80
**IOP (mmHg)**	17(15,19.5)	17(15,21.0)	0.81
**proptosis**	16.72±1.37	20.65±2.04	<0.001
**CAS**	1(1,1.5)	4(3,5)	<0.001
**Time of TAO (months) **	7.72±3.57	6.46±2.73	0.16
**TgAb(IU/ml)**	55.7±125.36	32.2±27.36	0.36
**TPOAb( IU/ml)**	60.1±105.28	51.5±73.72	0.74
** SBP (mmHg)**	122±8.11	123±5.09	0.49
**DBP (mmHg)**	80.72±6.41	79.92±4.34	0.65
**FBG(mmol/L)**		5.67±0.95	
**Axial length (mm)**	5.65±0.84	24.39±0.80	0.80
**body temperature(℃)** **history of smoking(Yes:no)**	36.66±0.45 8:17	36.58±0.42 6:20	0.60 0.54

*F* Female, *M* Male, *BCVA* Best-corrected visual acuity, *IOP* intra-ocular pressure, *CAS* clinical activity score, *TgAb* thyroglobulin antibody, *TPOAb* thyroid peroxidase antibody, *TAO* thyroid-associated ophthalmopathy, *SBP* Systolic Blood Pressure, *DBP* Diastolic Blood Pressure, *FBG* fasting blood glucose

*p < 0.05

OCTA and CDI were performed in all subjects. Based on quantitative analysis, the active group had higher choroidal OCTA parameters than those in the inactive group. The ChT, LA, and CVI in the active group were also significantly higher than those in the inactive group (*p* < 0.01), while the SA, TCA, and retinal OCTA parameters showed no significant difference between the two groups (*p* > 0.05).

Three vessels (OA, CRA, and PCA) were successfully and presented significant changes in CDI parameters between the two groups. The PSV and EDV of PCA significantly increased in the active group compared to the inactive group. However, there was no significant difference in the CDI parameters of OA and CRA, except for the PSV of CRA (10.29 ± 1.89 vs 11.56 ± 2.20, *p* = 0.03) (Table 2).

**Table 2:** Retinal, choroidal and retrobulbar parameters in TAO patients based on OCTA and CDI scan

Variables	inactive group	active group	**95% CI**	***P* value**
**Retinal parameters**				
SVD(%)	0.26 ± 0.06	0.27 ± 0.09	-0.03794 to 0.04843	0.81
DVD(%)	0.35 ± 0.04	0.34 ± 0.08	-0.04776 to 0.02684	0.56
**Choroidal parameters**				
ChT(um)	286.8 ± 71.25	439.8 ± 60.07	115.9 to 190.0	< 0.001*
TCA(mm2)	0.84 ± 0.35	1.02 ± 0.40	-0.02714 to 0.3949	0.08
LA(mm2)	0.43 ± 0.13	0.75 ± 0.16	0.2428 to 0.4029	< 0.001*
SA(mm2)	0.32 ± 0.10	0.31 ± 0.12	-0.07465 to 0.05129	0.71
CVI(LA/TCA)	0.59 ± 0.29	0.87 ± 0.53	0.03886 to 0.5185	0.02*
**Retrobulbar parameters**				
OA PSV(cm/s)	26.2 ± 9.93	30.61 ± 13.08	-2.144to10.97	0.18
OA EDV(cm/s)	7.39 ± 3.27	8.01 ± 2.52	-1.022to2.253	0.45
OA PI	1.08 ± 0.46	1.09 ± 0.37	-2.181to0.2490	0.89
OA RI	0.71(0.51,0.85)	0.73(0.64,0.80)	-0.1068 to 0.1207	0.90
CRA PSV(cm/s)	10.29 ± 1.89	11.56 ± 2.20	0.1139to2.425	0.03*
CRA EDV(cm/s)	3.42 ± 1.07	3.65 ± 1.30	-0.4432to0.9035	0.49
CRA PI	1.01 ± 0.25	1.06 ± 0.28	-0.09845 to 0.1985	0.5
CRA RI	0.66 ± 0.11	0.68 ± 0.11	-0.04501 to0.08326	0.55
PCA PSV(cm/s)	16.23 ± 4.82	19.93 ± 3.50	1.337to6.061	0.003*
PCA EDV(cm/s)	6.23 ± 2.65	8.14 ± 3.75	0.07788 to 3.743	0.04*
PCA PI	1.00 ± 0.28	0.73 ± 0.36	-0.4505 to -0.08732	0.004*
PCA RI	0.67 ± 0.11	0.53 ± 0.25	-0.2510 to -0.03198	0.01*

*SVD* superficial vessel density, *DVD* deep vessel density, *ChT* choroidal thickness, *TCA* total choroidal area, *LA* luminal area, *SA* stromal area, and *CVI* choroidal vascularity index, *OA PSV* ophthalmic artery peak systolic velocity, *OA EDV* ophthalmic artery end diastolic velocity, *OA PI* ophthalmic artery pulsatile index, *OA RI* ophthalmic artery resistance index, *CRA PSV* central retinal artery peak systolic velocity, *CRA EDV* central retinal artery end diastolic velocity, *CRA PI* central retinal artery pulsatile index, *CRA RI* central retinal artery resistance index, *PCA PSV* posterior ciliary artery peak systolic velocity, *PCA EDV* posterior ciliary artery end diastolic velocity, *PCA PI* posterior ciliary artery pulsatile index, *PCA RI* posterior ciliary artery resistance index,

* p < 0.05

To determine the relationship between CAS score and the OCTA and CDI variables, we ran a logistic regression model, for which the results suggested that only PSV(PCA) was significantly correlated with CAS difference (OR 1,847, 95%CI 1.145 to 2.979). None of the OCTA parameters showed any significant association with differences in CAS, nor did any other retrobulbar parameters. In other words, the greater the difference in the CAS scores between the two groups, the greater the difference in PSV(PCA) (Table 3).

**Table 3:** Logistic regression analysis between CDI parameters of PCA and CAS score

**variables**	OR	95%CI	*P* value
PCA-PSV	1.847	1.145to2.979	0.01*
PCA-EDV	0.408	0.141to1.182	0.09
PCA-RI	0.00	0.00to3.093	0.06
PCA-PI	0.005	0.00to1.629	0.07

*PCA PSV* posterior ciliary artery peak systolic velocity, *PCA EDV* posterior ciliary artery end diastolic velocity, *PCA RI* posterior ciliary artery resistance index, *PCA PI* posterior ciliary artery pulsatile index

^*^p < 0.05

The results of the correlation analysis between CDI parameters and OCTA parameters suggested that both LA and CVI were significantly correlated with PCA parameters, except for the PSV of PCA. In terms of retinal vascularity parameters, only SVD was inversely correlated with the RI of PCA (*r* = -0.27, *p* = 0.049, Table 4). However, there was no significant correlation between the OA and CRA CDI parameters, nor the retinal and choroidal vascularity parameters between the two groups (*P* > 0.05) (Table 4).

**Table 4:** Retrobulbar parameters in relation to retinal/choroidal vascularity

Variables	**Choroidal vascularity parameters**				**Retinal parameters**	
	TCA	LA	SA	CVI	SVD	DVD
OA-PSV	-0.1	0.09	-0.07	0.07	0.09	0.06
OA- EDV	0.03	-0.02	0.05	0.06	-0.02	-0.002
OA-RI	-0.08	0.16	-0.09	0.19	0.09	-0.08
OA-PI	-0.10	0.12	-0.11	0.15	0.07	-0.07
CRA-PSV	-0.004	0.09	-0.12	0.01	0.08	-0.16
CRA-EDV	-0.04	0.11	-0.08	0.18	0.11	0.16
CRA-RI	0.004	-0.02	-0.03	-0.13	-0.07	-0.27
CRA-PI	0.004	-0.02	-0.04	-0.12	-0.07	-0.25
PCA-PSV	0.00	0.18	-0.08	-0.09	-0.09	-0.05
PCA-EDV	0.03	0.33*	-0.14	0.31*	0.09	0.008
PCA-RI	-0.06	-0.42*	0.14	-0.32*	-0.27*	0.15
PCA-PI	0.02	-0.41*	0.25	-0.44*	0.07	-0.13

*TCA* total choroidal area, *LA* luminal area, *SA* stromal area, and *CVI* choroidal vascularity index, *SVD* superficial vessel density, *DVD* deep vessel density, *OA PSV* ophthalmic artery peak systolic velocity, *OA EDV* ophthalmic artery end diastolic velocity, *OA PI* ophthalmic artery pulsatile index, *OA RI* ophthalmic artery resistance index, *CRA PSV* central retinal artery peak systolic velocity, *CRA EDV* central retinal artery end diastolic velocity, *CRA PI* central retinal artery pulsatile index, *CRA RI* central retinal artery resistance index, *PCA PSV* posterior ciliary artery peak systolic velocity, *PCA EDV* posterior ciliary artery end diastolic velocity, *PCA PI* posterior ciliary artery pulsatile index, *PCA RI* posterior ciliary artery resistance index

^*^p < 0.05

Considering that proptosis is a severe complication of TAO, we performed a Pearson correlation analysis between the proptosis value and OCTA and CDI variables. OCTA parameters, including ChT (*r* = 0.05, *p* < 0.0001) and LA (*r* = 0.49, *p* = 0.002), were strongly associated with proptosis. In addition, CDI variables, including the PSV of PCA (*r* = 0.35, *p* = 0.01) and PI of PCA (*r* = -0.28, *p* = 0.04), were related to the proptosis value. Multivariate linear regression further indicated an association between the proptosis value and these variables. Among these variables, only choroidal vascularity parameters such as ChT (β = 0.009, *p* = 0.01), LA (β = 8.091, *p* = 0.01), SA (β = -11.2, *p* = 0.01), and CVI (β = -3.526, *p* = 0.04) were statistically correlated with proptosis. None of the retrobulbar CDI parameters showed any significant association with the proptosis value (*P* > 0.05, Table 5).

**Table 5:** Linear regression analysis evaluating association between all the variables and the proptosis value

Variables	Pearson correlation			Multivariate Linear regression		
	*r*	95%CI	*P*	β	95%CI	*P*
ChT	0.55	0.322to 0.716	< 0.0001*	0.009	0.002 to 0.017	0.01*
LA	0.49	0.256 to 0.679	0.002*	8.091	1.726 to 14.46	0.01*
SA	-0.17	-0.426to 0.109	0.23	-11.2	-19.70 to -2.850	0.01*
TCA	0.10	-0.1760to 0.369	0.47	-1.432	-5.164 to 2.300	0.44
CVI	0.19	-0.094to 0.438	0.20	-3.526	-6.828to -0.2241	0.04*
SVD	0.09	-0.190to 0.356	0.53	1.477	-6.623 to 9.576	0.71
DVD	0.08	-0.195to 0.351	0.56	10.27	-2.853 to 23.39	0.12
OA-PSV	0.07	-0.214to 0.334	0.65	0.081	-0.130to 0.292	0.44
OA- EDV	0.04	-0.238 to 0.311	0.78	-0.412	-1.236 to 0.411	0.32
OA-RI	-0.02	-0.289to 0.261	0.92	9.654	-13.18 to 32.49	0.39
OA-PI	-0.02	-0.290to 0.260	0.91	-8.038	-25.17 to 9.098	0.34
CRA-PSV	0.22	-0.063to 0.463	0.13	0.141	-1.152 to 1.434	0.82
CRA-EDV	-0.04	-0.313to 0.237	0.77	-0.101	-4.150 to 3.946	0.95
CRA-RI	0.18	-0.1023to 0.432	0.21	0.772	-71.35 to 72.89	0.98
CRA-PI	0.18	-0.105to 0.43	0.22	0.591	-23.93 to 25.11	0.96
PCA-PSV	0.35	0.081to 0.569	0.01*	0.186	-0.009 to 0.382	0.06
PCA-EDV	0.25	-0.025to 0.493	0.07	0.019	-0.363to 0.402	0.91
PCA-RI	-0.22	-0.468to 0.057	0.12	-2.682	-8.125 to 2.761	0.32
PCA-PI	-0.28	-0.516to -0.005	0.04*	-1.512	-4.681 to 1.658	0.34

*TCA* total choroidal area, *ChT* choroidal thickness, *LA* = luminal area, *SA* stromal area and *CVI* choroidal vascularity index, *SVD* superficial vessel density, *DVD* deep vessel density, *OA PSV* ophthalmic artery peak systolic velocity, *OA EDV* ophthalmic artery end diastolic velocity, *OA PI* ophthalmic artery pulsatile index, *OA RI* ophthalmic artery resistance index, *CRA PSV* central retinal artery peak systolic velocity, CRA EDV central retinal artery end diastolic velocity, *CRA PI* central retinal artery pulsatile index, *CRA RI* central retinal artery resistance index, *PCA PSV* posterior ciliary artery peak systolic velocity, *PCA EDV* posterior ciliary artery end diastolic velocity, *PCA PI* posterior ciliary artery pulsatile index, *PCA RI* posterior ciliary artery resistance index

*p < 0.05

## Discussion

To the best of our knowledge, alterations in retrobulbar blood flow, retinal microvasculature, and choroidal vascularity in patients with TAO have never before been examined simultaneously. Our results showed that ChT, LA, CVI, and blood flow velocity of PCA were significantly higher in patients with active TAO than in those with inactive TAO. Additionally, of all the above variables, only the PSV of the PCA was closely correlated with the CAS score. Furthermore, we found that choroidal OCTA parameters, including ChT, LA, SA, and CVI, were closely correlated with proptosis. Overall, our results suggest that changes in choroidal vascularity and posterior ciliary artery circulation were consistent with the aggravation of TAO.

While systemic factors such as hypertension or diabetes does not directly cause TAO, both of them closely intertwined with thyroid disease and can significantly influence the progression and symptoms of thyroid eye disease [16]. In this study, we did not find significant difference between the two groups in SBP, DBP and FBG values, those factors may not deeply correlate to disease activity owing to small sample size and absence of follow-up data. Although smoking might be a significant modifiable risk factor for development and progression of TAO [11], the proportion of smokers was not high at 32% and 23% respectively in both groups of our study population.

The significance of thyroglobulin antibodies and thyroid peroxidase antibodies in Graves' disease remains equivocal. Previous studies have described the roles of TG-Ab and TPO-Ab in the pathogenesis of Graves' disease (GD). Studies have shown that patients positive for TG-Ab with GD have lower TRAb titers and are in remission earlier than those negative for TG-Ab, Patients positive for TPOAb with GD have higher TRAb titers and need a longer time to achieve remission [17]. Other studies indicate that positive TPO-Ab and high Free triiodothyronine level in children with Graves' disease can be predictive factors for the presence of thyroid associated ophthalmopathy [18]. Our results showed that there were no difference in TPO-Ab and TG-Ab levels between the two groups. Thus, confounding factors caused by inconsistent levels of the two antibodies were excluded.

Several previous studies have reported similar choroidal changes in patients with TAO. For example, Bruscolini et al. [19] reported a significant increase in choroidal thickness in patients with Graves’ orbitopathy (GO), which was positively correlated with both CAS score and proptosis value. Çalışkan et al. [20] analyzed choroidal thickness in active GO, inactive GO, and healthy subjects, identifying a close association between choroidal thickness and CAS score. Thanks to the technological advances in OCT scanning, the EDI mode acquisition has improved the high-resolution visualization of the choroid [21]. However, choroidal thickness (CT) measurements exhibit intrinsic limitations with their dependence on gender, age, and refractive error [22]. Recently, a new OCT index, termed the choroidal vascularity index (CVI), has gained increasing interest in investigating the choroidal vasculature. CVI has good reliability for delineating the choroid with an image binarization process and is less influenced by physiological parameters [23, 24]. Loiudice et al. [25] previously reported that LA and CVI were significantly higher in TAO patients than in healthy controls. However, they found no association between choroidal thickness and the choroidal vascularity index and clinical parameters between the two groups. Yeter et al. [26] identified the proportional increases in LA, SA, and TCA in TAO groups, but found no statistically significant difference in CVI between the groups. In line with these previous studies, our study revealed that, in addition to ChT, LA and CVI were significantly higher in the eyes of patients with active TAO than that in the control group. LA was defined as the choroidal luminal (vascular) area, and SA was defined as the choroidal stromal (interstitial) area [23]. The pathogenesis of the acute stage is known to involve an inflammatory response as well as superior orbital vein congestion; as such, the increase in LA would be expected to be greater than the increase in SA in TAO patients [26]. Our data confirmed this hypothesis and revealed a significant increase in LA in the active TAO group versus the inactive group, while we found no statistical difference in SA between the two groups. Correlation analysis showed that ChT and LA were positively related to the proptosis value. We further found that the choroidal parameters ChT, LA, SA, and CVI, but not TCA, were statistically associated with proptosis. However, we found no significant correlation between choroidal parameters and CAS score, which is in contrast to the results of Noce et al. [1], who identified a strong correlation between choroidal thickness and CAS score. Some researchers have proposed that the choroidal infiltration of inflammatory cells, osmotically active molecules and fluid, increased vascular leakage, and congested choroidal vessels may lead to structural changes in choroid thickening [4, 27]. Taken together, these findings suggest that choroidal parameters such as ChT, LA, SA, and CVI could be regarded as sensitive risk predictors for the development of TAO.

The choroid, which serves as the major retinal blood supply, is composed of a complex vascular network with five main layers [28]. The posterior ciliary arteries contribute to the blood supply to the choroid vasculature, which originates from the ophthalmic artery [29]. Color Doppler imaging (CDI) is a noninvasive ultrasound method that has been successfully used to examine the characteristics of orbital hemodynamics in a variety of orbital disorders, including TAO [12, 30]. However, alterations in retrobulbar blood flow parameters, such as the posterior ciliary artery (PCA), central retinal artery (CRA), and ophthalmic artery (OA) exhibited significant variations, and previous studies have found conflicting data. According to Jamshidian-Tehrani et al. [31], OA-RI was significantly lower, and OA EDV was significantly higher in patients with thyroid eye disease (TED). However, this difference in CRA parameters did not reach statistical significance. Similarly, an analysis of 44 Graves’ disease (GO) cases found no statistically significant differences between the GO group and the controls in the flow parameters in CRA and OA [32]. In our study, the CDI parameters of the PCA were significantly different between the active and inactive TAO groups. In terms of OA and CRA parameters, only the PSV of the CRA exhibited a statistical increase in the active TAO group. The logistic regression model in our study showed that only PCA-PSV was an independent factor affected by the CAS score. In addition to the above evidence, we further found a close relationship between PCA (EDV, RI, and PI) and choroidal (LA and CVI) parameters. Given that the PCA supplies blood and nutrition to the choroid, changes in PCA blood flow may be more closely associated with the degree of TAO than OA and CRA. This indicates that the quantitative analysis of retrobulbar flow may be a promising prognostic indicator and predictor of TAO.

The major limitation of this study includes its cross-sectional design and small sample size. Follow-up studies are required to confirm our findings. Furthermore, owing to technical limitations, we did not evaluate the superior ophthalmic vein flow rate by CDI, which is a limitation as this has been considered a good feature of TAO to assess disease activity.

## Conclusions

Our research is the first study to combine ocular CDI and OCT parameters to reveal TAO clinical features and to help improve the understanding of TAO pathophysiology; therefore, it could serve as a reliable research tool for long-term follow-up management.

## Data Availability

The data used in present study are available by corresponding author on request.
